# Polymeric Co-Delivery Systems in Cancer Treatment: An Overview on Component Drugs’ Dosage Ratio Effect

**DOI:** 10.3390/molecules24061035

**Published:** 2019-03-15

**Authors:** Jiayi Pan, Kobra Rostamizadeh, Nina Filipczak, Vladimir P. Torchilin

**Affiliations:** 1 Center for Pharmaceutical Biotechnology and Nanomedicine, Northeastern University, Boston, MA 02115, USA; pan.jiay@husky.neu.edu (J.P.); rostamizadeh@zums.ac.ir (K.R.); ni.filipczak@northeastern.edu (N.F.); 2 Zanjan Pharmaceutical Nanotechnology Research Center, Zanjan University of Medical Sciences, Zanjan 4513956184, Iran; 3 Laboratory of Lipids and Liposomes, Department of Biotechnology, University of Wroclaw, 50-383 Wroclaw, Poland

**Keywords:** polymeric nanoparticles, stimuli-sensitive polymers, co-delivery systems, synergistic effect, nucleic acid delivery, chemotherapy

## Abstract

Multiple factors are involved in the development of cancers and their effects on survival rate. Many are related to chemo-resistance of tumor cells. Thus, treatment with a single therapeutic agent is often inadequate for successful cancer therapy. Ideally, combination therapy inhibits tumor growth through multiple pathways by enhancing the performance of each individual therapy, often resulting in a synergistic effect. Polymeric nanoparticles prepared from block co-polymers have been a popular platform for co-delivery of combinations of drugs associated with the multiple functional compartments within such nanoparticles. Various polymeric nanoparticles have been applied to achieve enhanced therapeutic efficacy in cancer therapy. However, reported drug ratios used in such systems often vary widely. Thus, the same combination of drugs may result in very different therapeutic outcomes. In this review, we investigated polymeric co-delivery systems used in cancer treatment and the drug combinations used in these systems for synergistic anti-cancer effect. Development of polymeric co-delivery systems for a maximized therapeutic effect requires a deeper understanding of the optimal ratio among therapeutic agents and the natural heterogenicity of tumors.

## 1. Introduction

Cancer, next to heart disease, ranks as the second leading illness-related cause of death worldwide with a growing incidence and mortality. It is one of the most challenging-to-treat diseases due mainly to inefficient pharmacologically active agents and the complexity of cancer. To date, chemotherapy has been widely used and has been the most efficient and successful treatment method in clinical practice. However, there are three major issues limiting the therapeutic efficacy of chemotherapy. First, most of chemotherapeutic agents have poor solubility that leads to deficiencies like low bioavailability, rapid blood/renal clearance, and nonspecific targeting, with significant undesirable side effects on healthy tissues. Second, non-uniform biodistribution limits the localization of drugs at the tumor site and leads to consequent demands for higher doses that have unacceptable toxicity. Above all, genetic variations that control survival and apoptotic pathways are involved in the development of cancers. Targeting an individual pathway with conventional chemotherapy is often unsuccessful in eradicating all cancer cells and results in multidrug resistance (MDR) over the course of treatment.

Several alternative approaches to overcome these problems associated with traditional chemotherapy have been established. Much attention has been focused directly on drug combination approaches with the aims of more effective treatment and decreased side effects [1,2,3]. In general, combination chemotherapy involves the simultaneous administration of two or more drugs with non-overlapping toxicities and dissimilar mechanisms of action so as to inhibit multidrug resistance. Combination therapy can overcome the toxicity of single drug therapy by targeting various signaling pathways. Lately, combination therapy regimens have been intensively studied, and the results of clinical practice have demonstrated synergistic effects that are greater than the sum of the individual drug effects and less systemic toxicity associated with the delivery of lower drug doses.

However, combination regimens are still limited by a low rate of successful outcomes and significant side effects due to low bioavailability of drugs and their nonuniform biodistribution. To take advantage of possible synergy between drugs, they must attain effective molar ratios at the tumor sites that are often hard to reach by conventional administration methods due to differences in injection schedules, pharmacokinetic properties, metabolism, and non-uniform biodistribution.

One strategy for delivery of drugs to the tumor site at the desired molar ratio involves the merging of nanotechnology with pharmacology and thereby take advantage of the nanoscale structures that carry multiple drugs, allow tuning of drug release, and modify biodistribution and pharmacokinetic characteristics of chemotherapeutic agents [4,5]. Such co-delivery systems may be used to not only regulate the dosages and the ratio of chemotherapeutic agents at the tumor site. They may also improve the efficacy of anticancer drugs through enhanced water solubility of hydrophobic molecules, lower toxicity, and higher stability, which prolongs blood circulation time to enhance accumulation in tumor tissues. Further enhancement of therapeutic efficacy can be achieved by taking the advantage of stimuli-responsive drug delivery systems equipped with target-activated moieties [6,7].

Co-delivery systems ideally possess the potential for encapsulation of both hydrophobic and hydrophilic drugs. Platforms for co-delivery systems should be designed to carry both traditional chemotherapeutics and cell regulatory molecules, such as nucleic acids [6]. Although much progress has been made with nanotechnology-based co-delivery systems, there are several problems to be considered in formulating an ideal drug delivery system including those associated with encapsulating drugs with a variety of solubilities and physicochemical properties, elevating drug concentrations within tumor tissues and regulating their sequential drug release.

To date, considerable efforts have been made to develop nano-particulate co-delivery systems for combination chemotherapy [6,8]. Various nanocarriers have been investigated, including lipid nanoparticles [9], liposomes [10,11,12], dendrimers [13] and polymeric nanoparticles [14]. More attention has been paid to polymeric nanoparticles mainly because of their potential to carry both hydrophobic and hydrophilic drugs, favorable controlled drug release characteristics, low toxicity, high stability and a prolonged circulation time which ultimately enhances accumulation in tumor targets. 

Many research and review papers involving co-delivery of therapeutic agents by polymeric nanoparticles in cancer therapy have been published [15,16,17]. However, therapeutic agents are often administered separately instead of simultaneously using a polymeric nanoparticle capable of delivering both agents [18]. Additionally, to the best of our knowledge, there is no comprehensive review addressing the effects of the drug ratio in co-delivery systems, a likely significant parameter promoting a synergistic therapeutic effect. The scope of this review is distinct from generalizations of about polymeric co-delivery systems for chemotherapy. Our aim is to address the dosage, cell type, mechanism, and their efficacy relationships that need to be considered in designing co-delivery systems.

## 2. Polymer Types Used in Preparing Co-Delivery Systems

### 2.1. Block Co-Polymer Conjugates

Amphiphilic polymers self-assemble into nanoparticles that are ideal co-delivery systems for both hydrophilic and hydrophobic drugs (Table 1). These polymers are usually obtained by conjugating together polymers with diverse properties. The block co-polymers formed inherit the properties of each block, thus integrating advantages of various blocks into a single system. Depending on the chemical composition, block co-polymers are prepared by conjugating hydrophobic and hydrophilic polymers together through physical/chemical interactions or by modifying hydrophilic polymers with hydrophobic lipid moieties. These synthetic block co-polymers self-assemble into polymeric nanoparticles or micellar nanoparticles for co-delivery purposes. In order to minimize toxicity and side effects, the major materials used are biodegradable polymers: chitosan [19], poly(lactic acid) [20], gelatin [21], poly[*N*-(2-hydroxypropyl) methacrylamide] (HPMA) [22] and their copolymers, poly(lactide-co-glycolide-co-caprolactone) [23] and poly(lactic-co-glycolic acid) [24]. Their total degradation can occur in the body and can reach the kidney threshold for excretion [25]. 

Six classic methods for obtaining polymeric nanoparticles have been described: nanoprecipitation [26], emulsion-diffusion [27], emulsification-coacervation [28], double emulsification [29], surface polymerization [30], and layer-by-layer methods [31]. In addition, multifunctional polymeric structures with precisely defined morphology can be obtained by a controlled atom transfer radical polymerization (ATRP) method [32].

Polymeric systems are grouped below by sensitivity to changes in temperature, pH, light, redox potential, and other special factors in their environment. Stimuli-sensitive polymers have become one of the most prominent solutions for anti-cancer therapy. The unique properties of polymers allow them to change their accumulation and drug release profile depending on the surrounding conditions. They are used to target drugs, bioactive substances and genes. These systems selectively deliver therapeutic agents to target tissues, cells and cell compartments to release their cargos [33,34,35]. By doing so, the pharmacological properties, release profile and therapeutic outcomes are improved compared to delivery as free drugs.

### 2.2. Thermo-Sensitive Polymers

Polymeric nanoparticles formulated with thermo-sensitive polymers has been applied to activate and control the release of active ingredients after reaching the target site. For example, overheating a cancer by activating magnetic cargo-loaded polymeric nanoparticles with a local magnetic field [36]. Thermos-sensitivity has been one of the most commonly used stimulating features for biomedical applications [37]. There are two types of polymers distinguished by their phase distribution. The first type, called UCST (upper temperature of the critical solution), passes between phases during cooling. In the second type LCST (lower critical temperature of the solution), this transition occurs with increasing temperature. Systems with UCST are more prevalent for polymers soluble in an organic solvent, while systems with LCST exist mainly for polymers dissolved in aqueous solvents. The solubility of polymers in organic solvents is due to short-range van der Waals interaction. The solubility of polymers in water is related to hydrophobic and hydrophilic interactions and the formation of hydrogen bonds. For polymeric drug delivery systems, mainly aqueous solvents are used [38,39,40].

### 2.3. pH-Sensitive Polymers

The sensitivity of polymers to pH has been utilized in polymeric nanoparticles designed for drug administration via the gastrointestinal tract (pH ranges from 2–4 in the stomach to about 6.8 in the gut), in solid tumors where the interstitial space is more acidic due to hypoxia, with a pH of about 6.5 compared to a blood plasma pH of about 7.4. These pH-sensitive polymers can be weak bases (more polar in an acid environment in a protonated form) or weak acids (more polar in a basic environment in deprotonated form). Polymers abundant with primary amines are sensitive to low pH (pH 5.0) and can facilitate the endosomal escape of drugs. Additionally, pH differences between normal tissue and tumor tissue can create conditions for use of pH-sensitive polymeric drug delivery systems with enhanced targeting and reduced side effects [63,64].

### 2.4. Redox-Sensitive Polymers

The redox potential differences in the tumor microenvironment inspired the idea of building a redox reaction-sensitive polymeric system for cancer treatment. Redox sensitivity is usually used in cases where changes of redox potential occur in inflamed tissues compared to healthy tissues. Changes in redox potential in cancer tissues are due to the production of reactive oxygen species by activated macrophages. Oxidatively degradable polymers, such as arylborone based on acid esters (which after oxidation become phenols and boric acid), or dialkyl sulphide-based polymers (which after oxidation become more hydrophilic), have been used as delivery systems for drugs to inflamed tissues [65]. The disadvantage of these polymers is that the level of reactive oxygen species is often not enough to fully oxidize the polymer so that the drug/gene cannot be released [66,67]. Drug delivery systems also use polymers that react to light exposure and the presence of certain ions or organic molecules including sodium alginate [68]. These types of polymer are applied mainly for diagnostic purposes [69].

It can be said that designing a polymeric drug system with micro-environmentally sensitive polymers is a “smart” strategy. Combining multiple therapeutic agents that inhibit tumor growth through different pathways into one system is also a “smart” strategy. Many polymeric systems have shown promising effects in cancer therapy based on these two ideas.

## 3. Application of Polymeric Nanoparticles as Co-Delivery Systems

### 3.1. Polymeric Nanoparticles for Co-Delivery of Chemotherapeutics

**Figure 1:**
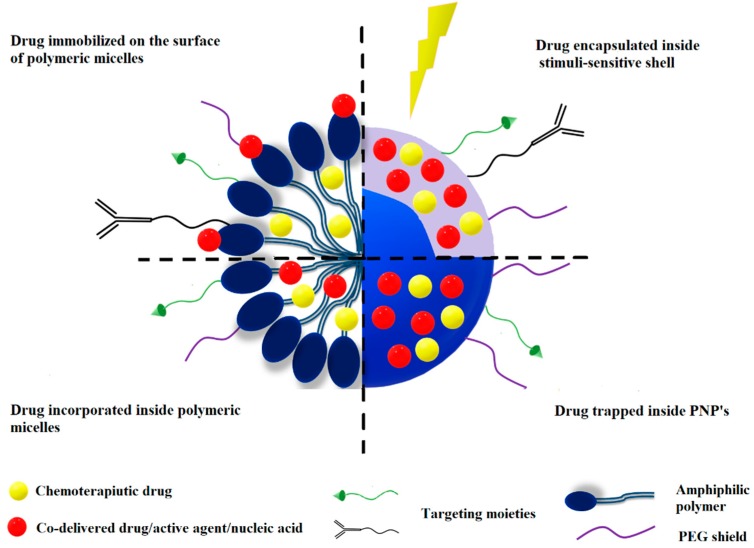
Drug loading in polymeric nanoparticles used as co-delivery systems in cancer treatment.

The aim of combining chemotherapeutics is to achieve an additive or even a synergistic effect. By targeting different pathways, combination therapy delivered by polymeric nanoparticles makes cancer cells more susceptible to the delivered therapeutic agents. Four commonly used approaches for co-delivery of therapeutic agents using polymeric nanoparticles are shown in Figure 1. Most drugs are passively loaded in polymeric nanoparticles according to their hydrophilicity. Hydrophobic molecules can be loaded in the hydrophobic moieties of micelles or polymersomes, and hydrophilic molecules are trapped in the hydrophilic compartments. Some drugs, such as nucleic acids, are co-loaded on the surface of the polymeric nanoparticles by electrostatic forces or chemical conjugations. Another approach is to directly conjugate drugs with the polymer through ester, amide or disulfide bonds [70]. Different types of polymeric nanoparticles can be further modified with targeting moieties.

Doxorubicin (DOX), a chemotherapeutic anthracycline, has been used clinically for treatment of several hematologic malignancies and solid tumors including breast cancer [71]. However, using DOX alone usually causes serious side effects in normal tissues, especially cardiotoxicity [72]. The molecular mechanism of DOX-induced cardiotoxicity is still unclear [73,74]. However, it has been postulated that it is caused by conversion of quinone into free radicals of half quinone, which in turn initiates cascading reactions leading to production of reactive oxygen and nitrogen in the body [75,76]. To increase the antitumor effect of DOX by overcoming cells resistance and, at the same time toning down the cardiotoxicity, DOX is often used in combination with other chemotherapeutics, nucleic acids and antibodies in cancer therapy [77,78].

The combination of DOX with other anticancer drugs aims to achieve a synergistic effect of the combined drugs or improve their biodistribution. Doxorubicin is often combined with Paclitaxel (PCT). The hydrophobic delivery systems containing PCT are based on the entrapment of the drug in their hydrophobic core. The combination of DOX and PCT in mPEG-PLGA polymeric nanoparticles made by a dual emulsion method has allowed a better anti-cancer effect in vivo and a much faster release of the drug from the carrier in an acidic environment [79]. A similar formulation was also used to destroy tumor stem cells [80]. The double-reacting nanoparticles built of four polymers approved by the Food and Drug Administration (FDA) for medical use were composed of poly (d-, l-lactide-co-glycolide) (PLGA), Pluronic F127 (PF127), chitosan, and hyaluronic acid (HA). Combining PLGA and PF127 forms a more stable and homogeneous nanoparticles than with PLGA or PF127 alone. HA was used as a targeting moiety towards cancer stem cells to reduce drug resistance associated with the dormant metabolic state. As a result of the combination of both drugs, the anti-cancer effect was amplified ~500 times compared with a simple mix of the two drugs [80]. Similar polymeric co-delivery systems were also used that enclosed the hydrophobic irinotecan (CPT) and hydrophilic DOX HCl, which inhibited the activity of topoisomerase I and II and exhibited an enhanced therapeutic effect on breast and brain tumors [81].

Another strategy, used to deliver camptothecin (CPT) and doxorubicin, was to form a graft copolymer with side drug segments that form nanostructures using a protein folding pathway [82]. The graft copolymer was constructed by direct polymerization of g-camptothecin-glutamate N-carboxyhydride (Glu (CPT)-NCA) at multiple sites on the PEG-based main chain via open ring polymerization (ORP). Only the conjugated CPT is hydrophobic and served as the main driving force during the assembly process. When exposed to water, the copolymer, together with DOX, curls to form monodisperse nanolayers for the delivery of the two drugs. PEG coated nanocarriers exhibit good stability and are internalized by various cancer cell lines through the lipid raft and clathrin-mediated endocytosis pathways without premature leakage. These nanolayers generated high synergistic activity of the CPT and DOX in various tumor cell lines. The in vivo study confirmed that the nanolayers can show strong accumulation at tumor sites and result in significant anti-tumor activity in a lung cancer xenograft model compared to free drugs [82].

**Figure 2:**
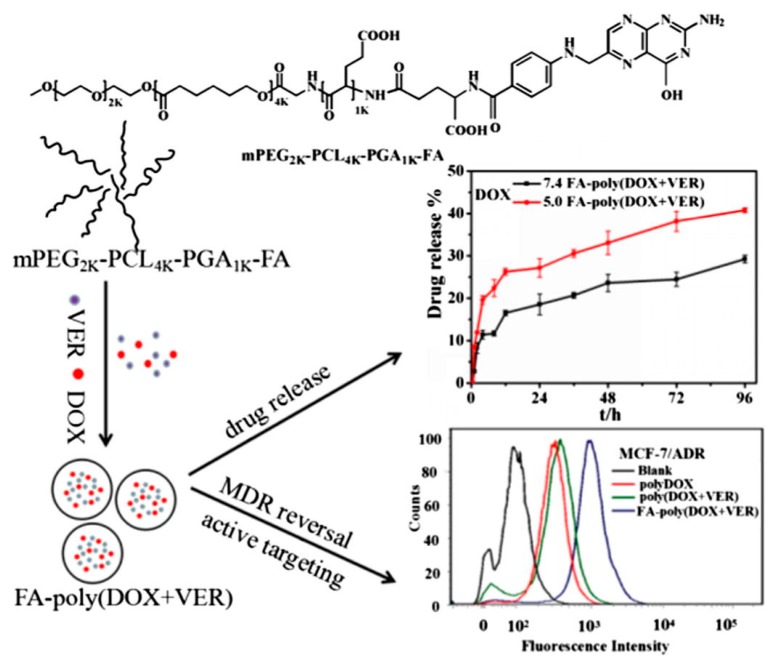
Folate-modified pH-sensitive co-delivery system of FA-poly(DOX+VER) polymer assembly exhibits obvious pH-sensitivity, high active targeting ability, strong multidrug resistance reversal and the enhanced therapeutic effect. Reproduced with permission from Li et al., Journal of Colloid and Interface; Elsevier, 2016 [83].

To overcome the drug resistance of tumors, redox-sensitive polymeric nanogels (<100 nm) based on poly(acrylic acid) were designed. Doxorubicin and cisplatin were enclosed in the nanogels by chelation, electrostatic interaction and π-π stacking interaction. Compare to free drugs, nanogels delivered more drugs to MCF-7/ADR cells. Considerable accumulation in cancerous tissues was observed in biodistribution experiments. In vitro anticancer studies showed better cell killing activity of the nano drug delivery system. All the data indicated that the combination therapy was more effective with reduced side effects [50]. Combinational delivery of DOX and verapamil in pH-sensitive polymeric nanoparticles based on co-polymer methoxy-poly(ethylene glycol)2k-poly(e-caprolactone)4k-poly(glutamic acid)1k (mPEG2k-PCL4k-PGA1k-FA) demonstrated a high drug release efficiency in tumor environment. The system was reported to overcome the multidrug resistance and improve the anti-cancer effect in MCF7/ADR cells (Figure 2) [83]. Other strategies to overcome the drug resistance of a tumor include regulation of the level of multidrug resistant protein [84]. Combining chemotherapeutics with gene therapy in polymeric nanoparticles for cancer treatment has received extensive attention [85,86].

### 3.2. Polymeric Nanoparticles for Co-Delivery of Nucleic Acid Therapeutics and Chemotherapeutics

Multiple genetic targets have been established for cancer treatment over the past several decades. Based on the genetic links associated with tumor progression and development, nucleic acid therapeutics, such as siRNA, plasmid DNA (pDNA), miRNA, and antisense oligonucleotides, have provided highly attractive approaches to downregulate tumor-associated proteins or to recover the function of tumor-suppressing pathways. However, because of their high molecular weight, large amounts of negative charge, and enzyme-induced degradation, nucleic acids are very unstable in the systemic circulation and can barely penetrate the cellular membrane. Thus, intensive efforts have been made to develop delivery systems for nucleic acid therapy [87]. Here, combinations of chemotherapeutics together with siRNA, pDNA, and miRNA delivered by polymeric nanoparticles for cancer treatment are discussed.

#### 3.2.1. Co-Delivery of siRNA and Chemotherapeutics with Polymeric Nanoparticles

One of the most widely used nucleic acid molecules used in preclinical and clinical studies has been small interfering RNA (siRNA). Cationic charged polymers, including chitosan [88,89], poly(ethylenimine) [90], poly(amidoamine) (PAMAM) dendrimer [13,91], and poly [2-(*N*,*N*-dimethyl aminoethyl) methacrylate] (PDMAEMA) [92,93], are capable of complexing with siRNA through electrostatic interaction, preventing degradation of siRNA and enhancing delivery of siRNA across the cell membrane. These polymers can be further modified by conjugating with other polymeric compartments to form an amphiphilic polymer with the ability to deliver siRNA and chemotherapeutics simultaneously.

Chitosan is a non-toxic and efficient vector for siRNA delivery. Surface modification with cationic chitosan by either absorption or covalent binding is a good strategy to enable the traditional drug delivery system to deliver siRNA as well [94]. Wang et al. coated chitosan on PLGA (50:50) nanoparticles loaded with DOX and grafted with a co-delivery system for both DOX and siRNA for an epidermal growth factor receptor [95]. Targeted with angiopep-2, the co-delivery system induced a 13% higher cell apoptosis rate than a PLGA nanoparticle loaded with DOX alone.

Cao et al. conjugated low molecular weight PEI with poly(ε-caprolactone) (PCL) through disulfide or ester covalent linkages [96]. This amphiphilic PEI-PCL self-assembled into a micellar structure. Doxorubicin was loaded into the PEI-PCL micelles using a chloroform/water mixture with sonication. Bcl-2 siRNA was complexed with PEI-PCL micelles followed by further modification with PEG chains to decrease the cytotoxicity of the nanoparticles. This DOX and siBcl-2 co-delivery system induced a 60% decrease in cell viability 96 h after treatment in a Bel7402 cell line. However, the cell viability decreased by only 40% when siScramble was used instead of siBcl-2, indicating a synergistic effect with the co-delivery of DOX and siBcl-2. With further modification of the DOX plus siBcl-2 loaded nanocarrier using folic acid, the cell viability decreased to 5%.

Another co-delivery system for siRNA and chemotherapy based on PEI was reported by Navarro et al. [97]. To prepare the amphiphilic molecule, PEI was conjugated with a dioleoylphosphatidylethanolamine (DOPE) moiety. Micellar nanoparticles formed by PEI-DOPE reversed the multidrug resistance in MCF7/ADR cells with delivery of P-glycoprotein siRNA (siP-gp) with DOX. Similarly, stearic acid was attached to PEI (1.8 kDa and 10 kDa) as a hydrophobic compartment by Huang et al. [98]. The combination of DOX and VEGF siRNA (siVEGF) co-delivered by PEI-SA micelles produced a promising in vivo Huh-7 tumor growth inhibition effect over 30 days. Tang et al. constructed an amphiphilic polymer, polyethyleneimine-block-poly((1,4-butanediol)-diacrylate-b-5-hydroxyamylamine) (PEI-PDHA), based on PEI 1.8 kDa [46]. Together with PEG-PDHA, this polymer self-assembled into nanoparticles co-loaded with siSnail, siTwist, and PCT. Significantly, an enhanced cytotoxic effect was observed at a PCT concentration of 50 µg/mL. The IC50 of the nanoparticles loaded with the three agents was 54.7-fold lower than that of free PCT.

PAMAM dendrimer is another candidate for siRNA delivery. The siRNA molecules are complexed via the primary amines on the surface of PAMAM dendrimers. Hydrophobic compartments used to load hydrophobic drugs can also be attached on the molecules through chemical conjugation. Biswas et al. prepared a tri-block co-polymer PAMAM-PEG2k-DOPE by conjugation between G4 PAMAM and DOPE-modified PEG [99]. PAMAM-PEG2k-DOPE self-assembled into micellar nanoparticles that complexed siRNA on the surface via the PAMAM moiety and encapsulated DOX base into the hydrophobic core. This co-delivery system efficiently delivered siRNA and DOX into cells and downregulated green fluorescent proteins (GFP) used to monitor the transfection efficacy in C166-GFP cells.

Another promising polymer for siRNA and chemotherapeutics co-delivery is PDMAEMA. Modification in the backbone of PDMAEMA not only decreased the cytotoxicity of the polymer, but also gave it the ability to load both siRNA and hydrophobic drugs [93,100]. Zhu et al. synthesized the block co-polymer PDMAEMA-PCL-PDMAEMA by free radical reversible addition-fragmentation chain transfer polymerization and assembled it into cationic micelles. PCT and siBcl-2 were delivered simultaneously using this system to PC3 cells, resulting in a 20% increased cytotoxicity than with free PCT after 24 h’ treatment [100]. Wang et al. also delivered siBcl-2 together with DOX using a PEG-PDEA-PDMA-DDAT triblock co-polymer. Nanoparticles loaded with both DOX and siBcl-2 increased the cytotoxicity by 27.5% and 19.8% compared to nanoparticles loaded only with Dox or only siBcl-2, respectively [41].

#### 3.2.2. Co-Delivery of pDNA and Chemotherapeutics with Polymeric Nanoparticles

Plasmid DNA (pDNA) was also delivered into cells after forming complexes with cationic polymers such as PEI, chitosan and PDMAEMA [101]. After modifying polymers with hydrophobic moieties, these polymers were widely applied for co-delivery of pDNA and hydrophobic chemotherapeutic agents. In 2006, Wang et al. developed PCT and interleukin-12 encoding pDNA co-delivery systems based on p(MDS-co-CES), which is poly{(*N*-methyldietheneamine sebacate)-co-[(cholesteryl oxocarbonylamido ethyl) methyl bis(ethylene) amoonium bromide] sebacate}. PCT was loaded inside the hydrophobic core during self-assembly, and pDNA was complexed with the cationic moieties. P(MDS-co-CES) micelles co-loaded with PCT and pDNA resulted in a greater tumor reduction than treatment with the gene or drug alone in a 4T1 mouse breast cancer model [102].

An abundance of primary amines makes branched PEI an ideal candidate for interaction with the large number of phosphate groups found on pDNA. Similar to the PEI-PCL used for co-delivery of siRNA and DOX, PEI1.8k-PCL was also reported as a co-delivery system for pDNA and hydrophobic drug by Qiu et al. [103]. After loading with both DOX and pDNA encoding luciferase reporter gene, PEI-PCL self-assembled into nanoparticles. Higher gene transfection efficacy than PEI25k and enhanced cytotoxicity compared to DOX alone were achieved in HepG2 [103]. Based on this structure, Shi et al. crafted a triblock co-polymer which consisted of mPEG5k-PCL2k-PEI2k for the delivery of DOX and Msurvivin T34A encoding pDNA [104]. By reducing the proliferation of tumor cells through Msurvivin T34A-induced caspase-mediated apoptosis, the author showed a higher anti-tumor effect compared to micelles loaded with DOX or pDNA alone. The author also suggested that treatment with dual-loaded micelles may allow lower doses of chemotherapeutics while maintaining a similar therapeutic outcome and help mitigate the toxic effect associated with high dose chemotherapeutics [104].

#### 3.2.3. Co-Delivery of miRNA and Chemotherapeutics with Polymeric Nanoparticles

MiRNAs regulate the multiple molecular pathways associated with cancer development with a high degree of biochemical specificity and potency [85]. Combination of miRNA with small molecule therapeutics has provided an unprecedented opportunity to improve therapeutic efficacy in a broad range of human cancers [105,106,107,108]. Using vectors identical for siRNA delivery, modified cationic polymers have been widely applied for co-delivery of miRNA and small molecule drugs. Mitall et al. conjugated gemcitabine (GEM) and complexed miR-205 mimics on a mPEG-b-PCC-g-GEM-g-DC-g-CAT co-polymer, which self-assembled into polymeric micelles. GEM and miR-205 mimics were co-delivered at concentrations of 500 nM and 100 nM in vitro. Co-delivery of both drugs in polymeric micelles reduced tumor cell migration and restored chemosensitivity to gemcitabine in resistant MIA PaCa-2R pancreatic cancer cells. Intratumor injection of miR-205 (1 mg/kg)/gemcitabine (40 mg/kg) micelles to mice bearing MIA PaCa-2R xenografts potently arrested tumor growth, whereas free gemcitabine or gemcitabine micelles had only a modest effect [109].

Hyaluronic acid-chitosan nanoparticles used to co-encapsulate DOX and miR-34a were reported by Deng et al. for treatment of triple negative breast cancer. Combinations of 100 nM of miR-34a and 0.1/0.5/2.5 µM of DOX were investigated for their cytotoxic effect on MDA-MD-231 cells. Nanoparticles loaded with both drugs significantly increased cytotoxicity at all three DOX concentrations. A superior in vivo efficacy of the combination therapy with 5 mg/kg of DOX and 2 mg/kg of miR-34a was further demonstrated in mice bearing MDA-MB-231 subcutaneous xenografts following intravenous administration of the co-delivery nanoparticles every two days [110]. Similarly, Wang et al. prepared hyaluronic acid coated PEI-PLGA nanoparticles as a polymeric co-delivery system for miR-542-3p and DOX. A range of DOX concentrations from 0.25 µg/mL to 2 µg/mL was investigated for their therapeutic efficacy with 100 nM of miR-542-3p. The highest cytotoxicity increase, compared to nanoparticles loaded with DOX only, was at a combination of 2 µg/mL of DOX and 100 nM of miR-542-3p in triple negative breast cancer cell lines [111].

**Figure 3:**
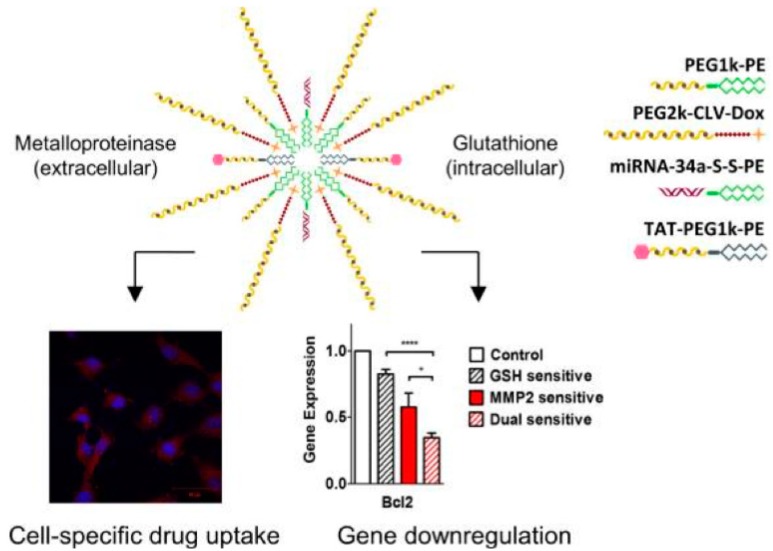
MMP-2 and glutathione sensitive polymeric nanoparticles used for co-delivery of DOX and miRNA-34a. Reproduced with permission from Salzano et al., Small; John Wiley and sons, 2016 [48]. * *p* ≤ 0.05, **** *p* ≤ 0.0001, *n* = 3, error bars represent mean ± SD.

In addition to nanoparticles composed of cationic polymers, neutral nanoparticles have also been used to co-encapsulate miRNA and chemotherapeutics. As reported by Salzano et al., miRNA-34a was conjugated with phospholipids through a disulfide bond, and DOX was conjugated with PEG through a metalloproteinase 2 (MMP-2)-sensitive peptide (Figure 3). Conjugates were formulated into dual-stimuli sensitive micellar nanoparticles that can simultaneously deliver DOX and miRNA-34a. The combination of both drugs reduced HT1080 cell viability to 40% and 50% in monolayer and 3D spheroids tumor models, respectively [48]. Liu et al. incorporated miR-200c and docetaxel (DTX) into the PEG-gelatinase cleavable peptide-poly(ε-caprolactone) (PEG-pep-PCL) nanoparticles. The concurrent delivery of 10 mg/kg of DTX and 10 mg/kg of miR-200c markedly potentiated the anti-tumor efficacy of DTX in vivo [112].

## 4. Dose/Efficacy Relationship within Co-Delivery Systems

Different combination delivery systems of anti-cancer drugs within a single polymeric vesicle have been discussed in previous sections. The aim of these polymeric system is to leverage the different mechanisms of the individual therapeutic agent for additive or synergistic therapeutic effects. However, eventual success is determined by several other important factors that deserve consideration in designing polymeric co-delivery systems. First, binding of pDNA onto polymeric nanoparticles affects the DOX loading and vice versa. Xu et al. reported that co-encapsulation of p53 pDNA and DOX within PLA coated PLGA microsphere resulted in significantly reduced encapsulation efficiency of DOX [113]. However, similar hurdles were less obvious in carriers loaded with RNA and DOX. Second, another factor that influences the therapeutic outcome can be ascribed to the drug release profiles of different payloads. Depending on the interaction between drugs and polymers, drug delivered by hydrophobic interaction may be released much faster than those conjugated with polymers under physiological conditions [114]. Thus, the drug release profile of polymeric nanoparticles is one of the most crucial factors to consider when designing polymeric co-delivery systems [79,115]. Third, due to different mechanisms of therapeutic agents, the therapeutic efficacy of one drug may disguise the effect of others. Zhang et al. screened the IC50 of DOX and curcumin (CUR) at different ratios using SMMC 7721 cells after 48 h treatments. The IC50 value for DOX and CUR in cells treated with a combination of DOX/CUR at ratio of 1:10 decreased to 0.46 µM and 4.65 µM, respectively, compared to IC50′s of free DOX at 1.30 µM and free CUR at 25.7 µM [115]. Much research requires a focus on optimizing the drug ratio for the optimal synergistic effect prior to co-loading multiple drugs into nanoparticles.

We would like to further elaborate on how drug combinations co-delivered are related to their therapeutic outcomes by concentrating on the dosing ratios between different therapeutic agents loaded in polymeric nanoparticles. Among the research published within the past decade, certain drug combinations have been widely used as models for developing co-delivery systems, including DOX, PCT, CUR, siBcl-2, siMDR-1. Although identical in drug combination, the therapeutic outcomes of polymeric co-delivery systems vary between cells and types of polymers. Here, we reviewed the factors that influence the therapeutic outcomes, with the aim of providing guidance in the design of polymeric co-delivery systems.

### 4.1. Dose Combinations of Chemotherapeutics in Polymeric Co-Delivery Systems

#### 4.1.1. Doxorubicin and Paclitaxel Combinations

Combining two chemotherapeutics in a single carrier has been a popular approach in designing polymeric co-delivery systems. To validate the efficacy of these systems, a lot of work has to been done to characterize the relationship between the therapeutic outcome with two agents and the ratio of the two agents. In Table 2, drug combinations, at which synergistic anti-cancer effect was achieved, were listed. DOX and PCT are two classic chemotherapeutic agents that have been applied extensively in various polymeric systems. Combination delivery of both DOX and PCT are attractive for their achievement of a higher therapeutic outcome. Wang et al. loaded hydrophilic DOX and hydrophobic PCT in methoxyl PEG-PLGA co-polymer nanoparticles. The mPEG-PLGA co-loaded with both DOX and PCT produced greater tumor growth inhibition in vitro than mPEG-PLGA loaded with either DOX or PCT at the same concentrations. Moreover, the highest anti-tumor efficacy was achieved when DOX and PCT were loaded at a concentration ratio of 2:1 using three different types of tumor cell line [79].

**Figure 4:**
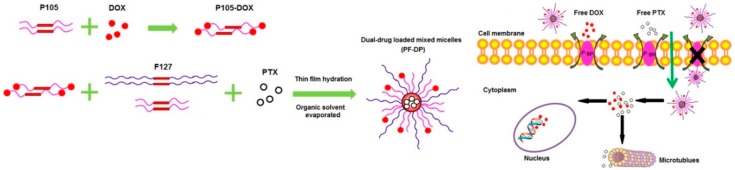
Co-delivery of DOX and PCT in polymeric nanoparticles consist of P105 and Pluronic F127 into MCF7/ADR cells. Reproduced with permission from Chen et al., International Journal of Pharmaceutics; Elsevier, 2015 [117].

Xu et al. prepared an amphiphilic poly(ethylene glycol)-poly(l-lactic acid) (PEG-PLA) diblock co-polymer and incorporated DOX and PCT into the ultrafine PEG-PLA fibers by an “emulsion-electronspinning” method [116]. The authors showed a lower cell viability and higher percentage of cell cycle arrest when DOX and PCT were delivered at a concentration ratio of 1:1 to rat glioma C6 cells 72 h after treatment. Chen et al. conjugated Pluronic P105 with DOX and encapsulated PCT into the hydrophobic core formed by P105-DOX and Pluronic F127 as a co-delivery system for hydrophobic DOX and PCT (Figure 4). The ratio between DOX and PCT was 2:3, when a higher in vitro cytotoxicity was observed in both MCF7/ADR and KBv cell lines. An increased apoptotic event, S phase cell cycle arrest and enhanced spheroid growth inhibition were observed in MCF7/ADR cells. The in vivo study also effected an efficient tumor growth inhibition over 14 days at this drug ratio [117]. Similarly, Ma et al. investigated the performance of pH-sensitive Pluronic F127-grafted chitosan for delivery of DOX together with PCT in vivo. In their study, 25 mg/kg of DOX and 20 mg/kg of PCT were used. All these studies used a concentration ratio between DOX and PCT of about 1:1 [118].

However, other groups have held a different opinion on the optimal drug ratio between DOX and PCT loaded in polymeric vehicles. Duong et al. also prepared a PEG-PLGA based co-polymer system for delivery of hydrophobic DOX base and PCT. In addition, folic acid and TAT peptide were included to enhance the cellular interaction between PEG-PLGA micelles and a human oral cavity carcinoma KB cell line. After screening several different combinations of DOX and PCT and calculated their effectiveness using Calcusyn software, they demonstrated a better synergistic effect of DOX and PCT at a concentration ratio of 1:0.2 than of a concentration ratio of 1:1 [119]. This ratio is supported by Lv et al.’s study. In their case, an amphiphilic deoxycholate decorated methoxy poly(ethylene glycol)-b-poly(l-glutamic acid)-b-poly(l-lysine) triblock co-polymer (mPEG-b-PLG-b-PLL) was synthesized and developed as a nano-vehicle for co-delivery of DOX and PCT. The DOX and PCT co-loaded nanoparticles at a concentration ratio of 4:1 showed an obvious synergistic effect. The CI50 value was approximately 0.57, indicating co-delivery of DOX and PCT had evident superiority in tumor growth inhibition as compared with free drug combinations [120]. In an animal study, 4 mg/kg of DOX and 1 mg/kg of PCT loaded in nanoparticles were given to animals every four days. An efficient tumor growth inhibition was observed over 18 days in a A549 xenograft tumor model. The tumor volume of the co-loaded nanoparticles-treated group was only 9.0% of the control group at the 18th day, which was 3.2-fold, 6.3-fold, and 2.4-fold smaller than when treated with free DOX, free PCT and free DOX + PCT, respectively [120].

Although various drug combinations were used among these different cases to achieve a synergistic effect, one conclusion shared by these results is that control of the amount of DOX used is a prominent factor. DOX has a faster release rate than PCT, and the release of DOX facilitates the release of PCT. No significant synergistic effect was observed when PCT was used in excess [119]. Additionally, PCT inhibited the tumor growth by stabilizing the microtubule during cell mitosis. Cells were arrested rather than entering an apoptotic pathway when treated with DOX. Thus, synergistic effects can hardly be observed 24–48 h after treatment because the effect of DOX overrode the effect of PCT [79].

#### 4.1.2. Doxorubicin and Curcumin Combinations

Another classis combination is DOX and CUR. CUR was believed to inhibit the tumor growth by causing cell cycle arrest [133,134], inducing an apoptotic signal [135,136], reversing multidrug resistance [137] and inhibiting the activation of NF-κB [138,139]. The exact molecular mechanisms of curcumin-induced apoptosis in cancer cells varied and depended on the cell type and dose used [140]. Application of CUR as an adjuvant in co-delivery systems has aroused great interest [141].

Zhang et al. combined pro-apoptotic, anti-angiogenic activities in pH-sensitive nanoparticles prepared with d-α-tocopherol poly(ethylene glycol)1k-block-poly (β-amino ester) (TPGS-PAE) polymers. The authors optimized the concentration of DOX and CUR at a 1:10 ratio. When exposed to 0.25 µM of DOX together with 2.5 µM of CUR, a 45% increased cytotoxicity occurred over that treated with DOX alone after 48 h in human SMMS 7721 liver cancer cells. The same ratio was also used in an in vivo study, where 1 mg/kg of DOX and 10 mg/kg of CUR were delivered within TPGS-PAE nanoparticles given intravenously once every other day. The CUR + DOX loaded nanoparticles induced a tumor weight suppression of 73.4%, compared to the 32.6% in a free CUR + DOX group [115].

Yan et al. also investigated the performance of a DOX CUR co-delivery system in a human liver cancer cell line, Hep G2. They came to a contradictory conclusion from Zhang’s. regarding the drug ratio between DOX and CUR needed to reach a synergistic effect [142]. They prepared a redox-responsive micelle composed of a glycyrrhetinic acid-modified chitosan-cystamine-poly(ε-caprolactone) co-polymer (PCL-SS-CTS-GA). DOX and CUR were loaded in the PCL-SS-CTS-GA nanoparticles at mole ratios of DOX:CUR = 1:1, 2:1 and 3:1. A synergistic effect was observed only at ratio of 2:1 and 3:1 in a Hep G2 cell line [142]. This result was similar to that reported by Zhang et al., who conjugated DOX with methoxy-poly(ethylene glycol)-aldehyde (mPEG-CHO) and encapsulated CUR into the hydrophobic core formed within the mPEG-DOX micelles. They used a 2.5-fold higher concentration of DOX compared to CUR corresponding to a mole ratio of DOX:CUR = 1.6:1. In both Hep G2 and HeLa cell lines, an enhanced cytotoxic effect was observed in the mPEG-DOX-CUR nanoparticle-treated group compared to cells treated with DOX or CUR alone [143].

In other studies where CUR was utilized in an attempt to overcome multidrug resistance, Wang et al. studied the effect of co-delivery of DOX and CUR in mice bearing MCF7/ADR or 4T1 tumors. The polymeric micelles for DOX and CUR co-delivery were based on two diblock co-polymers, d-α-tocopherol polyethylene glycol succinate (TPGS) and PEG2k-DSPE. Cells were incubated with polymeric micelles containing both drugs at a 1:1 ratio (mole ratio: DOX:CUR = 1:1.6) for 48 h. Over a concentration range of DOX of 0.5 µg/mL to 40 µg/mL, the maximum cytotoxicity increase was observed at 10 µg/mL. An 18.3-fold increase of apoptotic events was also observed at 10 µg/mL of DOX in the group treated with polymeric micelles loaded with DOX and CUR compared with the one treated with DOX only. A formulation co-loaded with 5 mg/kg of DOX and 5 mg/kg of CUR injected intravenously every two days resulted in a significantly lower tumor volume after a 10-day treatment than those treated with either DOX or CUR alone [144]. Duan et al. co-encapsulated 0.12 µg/mL of DOX and 0.2 µg/mL of CUR (mole ratio: DOX: CUR = 1:2.6) in chitosan/poly(butyl cyanoacrylate) nanoparticles to reverse the multidrug resistance in MCF7 ADR cell lines. They reported that polymeric nanoparticles loaded with both drugs induced a higher cell growth inhibition and a significantly greater downregulation of MDR-1 protein at 48 h after treatment [145].

Overall, the mole ratio of CUR and DOX reported for these polymeric delivery systems had a broad range, suggesting that in the presence of multiple tumor inhibiting pathways, CUR acts differently depending on the cell line. Thus, it’s hard to predict an effective universal ratio for combination treatment with CUR plus DOX. Additionally, the release profile of CUR differs markedly between polymeric delivery systems [146,147]. In some cases, DOX is directly conjugated with the polymer, while in other cases, DOX and CUR are both passively encapsulated. The release profile and types of tumor are key points to be taken into consideration during the design of polymeric co-delivery systems using CUR.

#### 4.1.3. Paclitaxel and Cisplatin Combination

The use of a combination of PCT and cisplatin (CDDP) is another classic and popular co-delivery strategy. Cisplatin, a member of the platinum-containing anticancer drugs, has been used intensively for the treatment of various solid tumors, particularly in advanced stages. There are different mechanisms that account for the effect of CDDP. It is well-known as a non-specific DNA-modifying agent that induces cell apoptosis by interaction with nuclear DNA to inhibit the transcription and replication of DNA. The clinical application of CDDP is limited because of its high dose-dependent toxicity, drug resistance, and low bioavailability. PCT, on the other hand, a number of the taxane family, works as a microtubule-stabilizing chemotherapeutic agent. It is known that PCT can also inhibit platinum−DNA adduct repair and enhance apoptosis of cisplatin-resistant tumor cells [148]. However, it enhances the nephrotoxicity of CDDP. Nowadays, the combination therapy including CDDP and PCT is still a popular clinical regimen used as a the first-line chemotherapeutic agent for advanced cancer treatment.

To date, few works have dwelt on the study of co-delivery of the CDDP/PCT, due mainly to the hydrophilic nature of CDDP and hydrophobicity of PCT, which has made the loading step very challenging [149,150]. One strategy to overcome this problem is to use different cisplatin prodrugs to facilitate drug loading [151,152]. Polymeric nanoparticles, particularly micelles with the ability to carry both hydrophilic and hydrophobic drugs represent a promising candidate for the co-delivery of CDDP and PCT. Mi et al. used a d-α-tocopherol-co-poly(ethylene glycol)1k succinate (TPGS)-cisplatin prodrug (TPGS-CDDP) along with docetaxel to improve CDDP loading into Herceptin-decorated PLA-TPGS nanoparticles. The highest drug loadings achievable for CDDP, PCT, and Herceptin using this approach were 3.5 ± 0.1, 9.0 ± 0.5, and 73.1 ± 5.8 µg/mg, respectively [151]. The presence of polymers with the ability to form a chelate with CDDP, such as PEG or poly-glutamic acid in the copolymer structure, can also enhance drug loading capacity [125,129,130]. In He et al.’s work, the drug loading capacity of PCT and CDDP attainable with FA-PLGA-PEG micelles has been reported to be 5.83 ± 0.04%, 4.68 ± 0.07%, respectively, at a polymer/drug ratio of 40:2:4 (polymer:CDDP:PCT) [153].

It is important to note that, the ratio of drug loading in the carrier plays a determinant role in achieving the highest therapeutic efficacy for combination therapy. According to Wan et al., the actual drug ratios in the tumor did not differ significantly from the drug ratios in the initial co-loaded drug formulations [129]. Thus, assuring the drugs loading for polymeric nanoparticles in an appropriate ratio would be of great importance in formulating a treatment. He et al. showed that in A549 and M109 cells, the effectiveness of co-delivery of NPs, with a CDDP/PCT concentration ratio of 1:2, was approximately twice that of CDDP [153]. Moreover, co-delivery of NPs, with a CDDP/PCT concentration ratio of 1:2, exhibited the greatest anti-tumor activity among the two varieties of lung cancer cell. Although in general, dual drug-loaded nanoparticles with a higher ratio of PCT exhibited the greatest response, there are some reports indicating the best response corresponds to an equal drug ratio [152].

Time-dependency is another factor affecting the synergism of CDDP/PCT co-delivery systems [125]. This phenomenon was related to the release behavior of CDDP and PCT from the carrier. In single drug-loaded nanoparticles, drug is quickly released, while in a dual drug-loaded carrier system, chelation of PCT with CDDP prevented an initial burst release and resulted in a lower growth inhibition effect. However, over time, more PCT was released, and the combination effect became manifest. The in vivo studies on co-delivery systems for CDDP and PCT also clearly revealed improved pharmacokinetics and biodistribution in the blood and tumor of either one or both drugs compared to single drug micelles [129].

### 4.2. Dose Combinations of Nucleic Acid Therapeutics and Chemotherapeutics in Polymeric Co-Delivery Systems

The rationale for combination of nucleic acid therapy with chemotherapeutics in a single platform to ensure that cells will be simultaneously exposed to two types of damage has been discussed previously. The dose combination between nucleic acids and chemotherapeutics delivered has large effects on the synergistic anti-cancer efficacy of such co-delivery systems (Table 3). Nucleic acid therapy induces tumor inhibitory effects via different molecular pathways than traditional chemotherapeutics. Plasmid DNA requires delivery into the nucleus followed by translation into therapeutic proteins and the oligonucleotide requires interference with the mRNA or proteins in various pathways to generate their effect. Thus, nucleic acid therapy takes a longer time than chemotherapeutics before the tumor growth inhibition effect can be observed. The effect of chemotherapeutic agents can also disguise the one from nucleic acid therapy. Therefore, controlling the ratio between nucleic acid molecules and chemotherapeutics involving different mechanisms and types of pathways in polymeric systems is critical for an ideal synergistic effect.

#### 4.2.1. Inducing Apoptosis through Delivery of TRAIL

Interfering with the apoptotic pathway by either enhancing the pro-apoptotic effect or inhibiting the anti-apoptotic effect with nucleic acid therapies is a promising cancer treatment. One of the most attractive pro-apoptotic pathways for the treatment of cancer is through the tumor necrosis factor (TNF)-related apoptosis-inducing ligand (TRAIL). TRAIL is a potent stimulator of apoptosis, and tumor cells are significantly more sensitive to TRAIL-induced apoptosis than normal cells [154]. However, numerous cancer cells exhibit a certain amount of resistance to TRAIL-induced apoptosis. Thus, combining TRAIL protein with traditional chemotherapeutics could increase the therapeutic efficacy in TRAIL-resistant cancer cells [155,156]. Lee et al. co-loaded both TRAIL protein and DOX in polymeric nanoparticles self-assembled from a biodegradable cationic co-polymer P(MDS-co-CES) to achieve a synergistic cytotoxic effect in cancer cells. A synergistic effect of a 40% enhanced cytotoxicity was observed with nanoparticles loaded with 10 nM of TRAIL and 0.8 µM of DOX compared to nanoparticles loaded with each drug alone in TRAIL-resistant SW480 colorectal carcinoma cells [157]. The same group also used P(MDS-co-CES) for co-delivery of TRAIL and PCT. In the study, various human breast cancer cell lines were exposed to 10 nM of TRAIL and 1.67 µM of PCT co-loaded in P(MDS-co-CES) polymeric nanoparticles. A 25% enhanced cytotoxic effect was achieved compared to single drug-loaded treatments [158].

In addition to delivering TRAIL in polymeric nanoparticles, Fan et al. delivered plasmid DNA encoded for TRAIL together with DOX in β-cyclodextrin-polyethyleneimine (PEI-CD) supramolecular nanoparticles (SNP). The PEI-CD SNP loaded with 0.5 µg/mL of DOX and 2.5 µg/mL of TRAIL pDNA (pTRAIL) induced many more apoptotic events than individual drug treatment in SKOV-3 cells after 48 h treatment. At the same drug ratio, SKOV-3 ovarian tumor-bearing mice received a combination treatment of 6 µg of DOX and 30 µg of pTRAIL twice per week. After 18 days, a significantly higher tumor growth inhibition was observed in those mice [47]. In another study, DOX was complexed with pORF-hTRAIL, which was then complexed with polyamidoamine-PEG-T7 (PAMAM-PEG-T7) through electrostatic interaction. A significant synergistic effect occurred both in vitro and in vivo when 12.5-fold more DNA than DOX by weight was loaded into this platform. Each Bel-7402 tumor-bearing mouse was treated with 50 µg of DNA in combination with 4 µg of DOX [159].

#### 4.2.2. Increasing Apoptosis by Restoring p53

A similar combination was also used to restore the function of the tumor suppressor, p53. Xu et al. treated Hep G2 cells with a combination of 2 µg/mL of p53 gene and 0.9 µg/mL of DOX. Chitosan-p53 nanoparticles and DOX molecules were co-loaded in double-walled microspheres that consisted of a PLGA core surrounded by a poly(l-lactic acid) (PLA) shell. Overall, the combined DOX and p53 treatment enhanced cytotoxicity and increased activation of caspase-3 compared to either DOX or p53 treatment alone [160].

Li et al. used a combination of DOX and p53 plasmid at 3 µg/mL and 4 µg/mL, respectively, to induce a synergistic anti-cancer effect in breast cancer. Both drugs were loaded in micelles formed from a star-shaped polymer consisting of a cationic poly[2-(dimethylamino) ethyl methacrylate] (PDMAEMA) shell and a zwitterionic poly{*N*-[3-(methacryloylamino) propyl]-*N*,*N*-dimethyl-*N*-(3-sulfopropyl) ammonium hydroxide} (PMPD) corona that was grafted from a polyhedral oligomeric silsesquioxane (POSS)-based initiator. A high tumor cell apoptosis in MCF7 cells occurred in vitro and extensive tumor growth inhibition was observed over 28 days with 1.5 mg/kg of DOX and 2 mg/kg of p53 plasmid administrated every five days [161].

**Figure 5:**
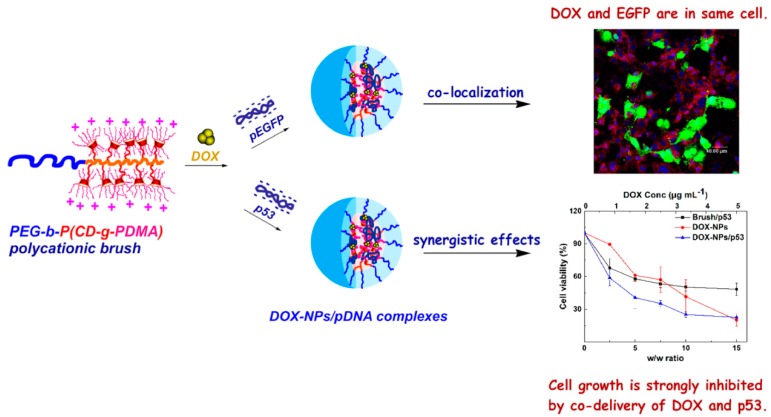
Co-delivery of DOX and pDNA in cationic polymeric nanoparticles with co-localization of cargos and enhanced tumor cell growth inhibition. Reproduced with permission from Chen et al., Polymers; MDPI, 2019 under the license CC BY 4.0 [173].

Usually, 2–4 µg/mL of plasmid DNA was used in co-delivery with chemotherapeutics to achieve a synergistic effect. But the ratio between chemotherapeutics and pDNA ranged from 1–10. Chen et al. investigated the response of cell viability in MCF7 cells to DOX-loaded polymeric nanoparticles complexed with different ratios of p53 pDNA (Figure 5) [173]. An enhanced cell growth inhibition was observed at weight ratios between DOX-NP and p53 pDNA from 5–10. This enhanced effect was not observed at weight ratios higher than 10 or lower than 5. These results suggested that the ratio between chemotherapy and pDNA for a synergistic effect could be affected by a diversity of cell lines and types of cationic polymers. Especially because that, as previously discussed, the complexation of pDNA with polymeric nanoparticles could affect the loading of chemotherapeutics [113]. Investigating the pDNA complexation effect on drug loading, drug release profile is essential in evaluating the anti-cancer performance of pDNA and chemotherapeutics-loaded polymeric co-delivery systems.

#### 4.2.3. Decreasing Anti-Apoptotic Effect through Downregulation of Bcl-2

Another popular candidate used within co-delivery systems with pro-apoptotic effects has been siBcl-2. Overexpression of Bcl-2 family proteins suppresses the cell death induced by chemotherapeutics [174]. Thus, it’s particularly interesting to pursue possible synergistic effects derived from the downregulation of Bcl-2 and administration of chemotherapeutics. Cao et al. proposed loading DOX and siBcl-2 into PEI-PCL micelles using a chloroform/water mixture under sonication. The micelles were then coated with folic acid-conjugated poly(ethylene glycol)-block-poly(glutamic acid) (FA-PEG-PGA) after complexation of siBcl-2. Together with 20 nM of siBcl-2, 0.05 µM of DOX was delivered to Bel-7402 liver cancer cells. The PEI-PCL/FA-PEG-PGA micelles co-loaded with DOX and siBcl-2 induced a 60% increased cytotoxicity 96 h after treatment [96]. From the same group, Cheng et al. investigated the therapeutic efficacy of PEI-PCL/FA-PEG-PGA micelles loaded with siBcl-2 and DOX using C6 glioma cells. They demonstrated that 25 nM of siBcl-2 achieved a higher knockdown effect than that at 12.5 nM. Most importantly, the knockdown effect of Bcl-2 became saturated at concentrations higher than 25 nM. The combined therapeutic outcome of DOX (22.5 µg/kg) and siBcl-2 (1.6 µg/kg) treatment in vivo highlighted the importance of combined therapy of DOX and siRNA for tumor growth inhibition [162].

Wang et al. also investigated the combination of DOX and siBcl-2 in another human liver cancer cell line, Hep G2, using a PDEA-PDMA-PEG co-polymer. They also used 20 nM of siBcl-2 but increased the DOX concentration to 1.69 µM. Under this circumstance, co-delivery of both drugs increased the cytotoxicity by 27.5% and 19.8% compared to nanoparticles loaded with DOX and siBcl-2, respectively [41]. Instead of DOX, PCT has also been delivered together with siBcl-2. Wang et al. studied the synergistic effect between siBcl-2 and PCT in triple negative breast adenocarcinoma MDA-MB-231 cells. In the presence of 20 nM siBcl-2, cell viability decreased from 78% to 59% and from 58% to 39% at PCT concentrations of 100 nM and 400 nM, respectively [102].

However, other groups have used a higher concentration of siBcl-2. For example, Zhu et al. treated PC3 human prostate cancer cells with a combination of 188 nM of siBcl-2 and 0.58 µM of PCT. The drugs were delivered using micellar nanoparticles composed of PDMAEMA-PCL-PDMAEMA triblock co-polymer. They reported a synergistic effect of about 20% increased cytotoxicity compared to free PCT at 24 h after treatment [100]. Such differences in the concentration of siBcl-2 used may have resulted from the responses to siBcl-2 seen in different cell lines. Instead of screening for the response of cells to siBcl-2, most groups have used a standard siRNA concentration of 100 nM in their studies [42,175].

#### 4.2.4. Decreasing the Anti-Apoptotic Effect through Downregulation of Survivin

Survivin is one of the most frequently occurring antiapoptotic proteins seen in cancerous tissues (i.e., breast, colon, pancreas, and lung). Its main mechanism of action depends on inhibition of caspase activation [176]. Through its action, survivin leads to increased proliferation of tumor cells [177]. Wang et al. developed a DOX, PCT, and survivin co-delivery system using a nano-emulsion composed of a methoxy-poly (ethylene glycol) block copolymer (mPEG-PLGA) and e-polylysine (EPL). The core of the nano-emulsion was DOX, and the PCT was enclosed in the hydrophobic layer. EPL on the surface of the nano-emulsion complexed siRNA by electrostatic interaction. Experiments in mice bearing a B16-F10 melanoma tumor showed a synergistic tumor growth inhibition effect from DOX (8.6 mmol/kg), PCT (17.2 mmol/kg), and survivin-siRNA (1.5 mg/kg) [178].

In Shi et al.’s report where the block copolymer mPEG-PCL-g-PEI was used for co-delivery of doxorubicin and Msurvivin T34A plasmid, a synergistic effect of DOX (4 mg/kg DOX) and Msurvivin T34A plasmid (5 mg/kg) was demonstrated in mice bearing a B16-F10 melanoma, both in subcutaneous and lung metastases models. Although they obtained only a slightly higher antitumor activity when compared to free DOX, they effectively reduced systemic toxicity of the treatment [104]. Survivin shRNA-encoding plasmid was also delivered to SKOV-3 cells by self-assembled supramolecular micelles composed of b-cyclodextrin-polyethylenimine (PEI600-CyD) and 2-amineadamantine-conjugated PCT (Ada-PCT) by Hu et al. They proved that simultaneously administrated PCT and shRNA at concentrations of 0.6 µg/mL, 2 µg/mL in vitro and 6 µg/animal, 20 µg/animal in vivo, respectively, induces significantly higher cell apoptosis and inhibits tumor growth [163].

Another co-delivery pluronic system P85-PEI/TPGS/PCT/shSur containing survivin hairpin RNA was developed to treat A549 human lung cancer. The purpose of this study was to overcome paclitaxel resistance. Simultaneously administrated PCT (10 mg/kg) and shSur (2 mg/kg) showed enhanced efficacy of anticancer activity including higher PTX-induced apoptosis and cells arrested in G2/M phase [168]. A combination of DOX and survivin shRNA was also investigated by Tang’s group for its effect in overcoming multidrug resistance. In their work, a pH-sensitive polymer based on poly(b-amino ester), poly[(1,4-butanediol)-diacrylate-b-5-polyethylenimine]-block-poly[(1,4-butanediol)-diacrylate-b-5-hydroxy amylamine] (PDP-PDHA) was synthesized. Nanoparticles containing 6 mg/kg DOX and 2 mg/kg shRNA were administered to MCF7/ADR tumor-bearing mice. The authors successfully raised the accumulation of DOX and shSur in the tumor tissue, resulting in a tumor growth inhibition of 95.9% after 21 days [169].

#### 4.2.5. Increasing Intracellular Drug Accumulation by Inhibiting Drug Efflux

P-glycoprotein (P-gp), encoded by the MDR-1 gene, overexpressed in many types of human cancers, contributes to the multidrug resistant effect. Downregulation of P-gp has been associated particularly with enhancing the therapeutic outcome of chemotherapeutics. Thus, it is a popular inclusion in co-delivery systems for its synergistic effect. Xiong et al. reported the co-delivery of siMDR-1 and DOX using polymeric micelles formed by poly(ethylene oxide)-block-poly(ε-caprolactone) (PEO-b-PCL) amphiphilic block co-polymers to improve the anticancer effect in the multidrug drug resistant human breast cancer cell line (MDA-MB-435/LCC6MDR1). Micelles containing 5 µg/mL of DOX and 100 nM of siMDR-1 led to a maximum of ~70% cell growth inhibition at 72 h after treatment [45]. Navarro et al. also demonstrated that the downregulation of P-gp led to the inhibition of DOX efflux activity resulting in an enhanced cytotoxicity of DOX in the MCF7/ADR cell line. The combination used in their study was 1 µg/mL of DOX and 100 nM of siMDR-1. Drugs were delivered in polymeric nanoparticles consisting of PEI modified DOPE [97].

Zhang et al. prepared polymeric micelles based on N-succinyl chitosan-poly-L-lysine-palmitic acid (NSC-PLL-PA) for co-delivery of DOX and siRNA targeting P-gp. The study revealed that the therapeutic efficacy was close to the maximum when the siRNA concentration reached about 100 nM. This finding indicated that 100 nM was sufficient to downregulate P-gp expression, increase intracellular DOX concentration, and maximize the therapeutic effects. The cytotoxicity results at 48 h after treatment also indicated that a synergistic effect was achieved at 5 µg/mL of DOX and 100 nM of siMDR-1. Increasing the concentration of DOX disguised the effect of siMDR-1, leading to a more than 80% cytotoxic effect among all groups. Additionally, micelles loaded with 0.5 mg/kg of DOX and 0.2 mg/kg of siMDR-1 given to tumor-bearing mice every three days showed a significant tumor growth inhibition over 24 days [164]. Other groups also reported synergistic effects derived from co-delivery of siMDR-1 with DOX using polymeric nanoparticles at similar concentration combinations. For example, Xu et al. co-delivered 3 µg/mL of DOX and 100 nM of siP-gp in polymeric vehicles prepared from triblock copolymers, folate/methoxy-poly(ethylene glycol)-block-poly(l-glutamate-hydrazide)-block-poly(*N*,*N*-dimethylaminopropyl methacrylamide) (FA/m-PEG-b-P(LG-Hyd)-b-PDMAPMA) to MCF7 breast cancer cells [52]. And Misra et al. overcame multidrug resistance in cells by co-delivering 11.6 µg/mL of DOX and 100 nM of siMDR-1 using dimethyldidodecylammonium bromide (DMAB)-coated PLGA nanoparticles in the MCF7 ADR cell line [165]. All these studies demonstrated the enhanced therapeutic efficacy against cancer using polymeric co-delivery systems.

**Figure 6:**
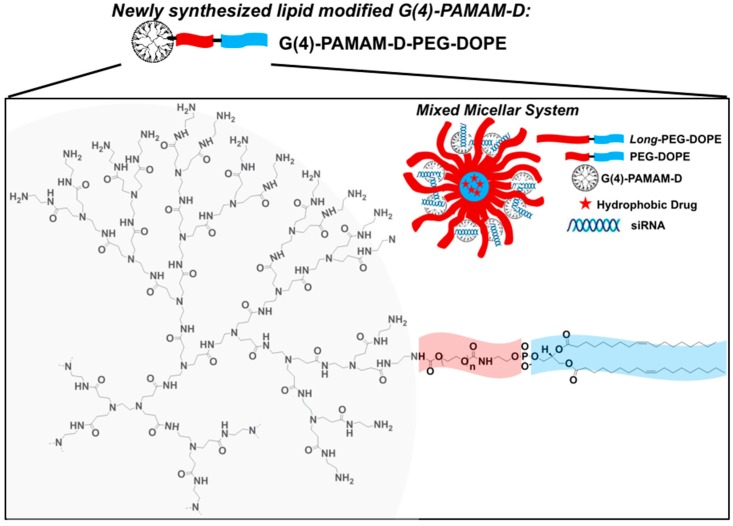
Schematic structure of mixed dendrimer micelles composed of PAMAM-PEG2k-DOPE and PEG5k-DOPE in co-delivery of DOX and siMDR-1. Reproduced with permission from Pan et al., European Journal of Pharmaceutics and Biopharmaceutics; Elsevier, 2019 [91].

In our previous work, micellar nanoparticles consisting of PAMAM-PEG2k-DOPE and PEG5k-DOPE were investigated as a co-delivery system for both hydrophobic drugs and siRNA (Figure 6). Combinations of DOX and siMDR-1 at different concentrations were applied to multidrug resistant cell lines A2780/ADR and MCF7/ADR [91,99]. The synergistic effect of the co-delivery system was observed in MCF7/ADR and A2780/ADR when treated with 125 nM of siMDR-1, 0.43 µM of DOX, or 125 nM of siMDR-1, 1.7 µM of DOX, respectively. The cytotoxicity results also suggested that co-delivery of siMDR-1 and DOX achieved an increased anti-cancer effect when delivery of siMDR-1 was followed by DOX treatment separately. Since downregulation of P-gp alone does not cause significant tumor growth inhibition, delivery of siMDR-1 together with intracellular delivery of chemotherapeutics is required for an ideal therapeutic effect. However, inadequate downregulation amount of P-gp or excess of chemotherapeutics could impair the performance of co-delivery systems targeting inhibition of drug-efflux. An optimal concentration ratio should be established for desired therapeutic outcomes.

#### 4.2.6. Inhibiting Tumor Growth by Altering Immune Responses

Among many strategies for co-delivery systems, being able to modulate the natural immune response against cancer cells is one of the most promising challenges. Chen et al. designed a co-delivery system of DOX + IL-36γ/POEG-st-Pmor with an improved anti-metastatic effect in a mouse breast cancer lung metastasis model with 4T1.2 cells. DOX 5 mg/kg and IL-36γ plasmid 50 μg per animal synergistically enhanced the immune response (type I) by increasing the IFN-γ positive CD4+ and CD8+ T cells [171]. Wu et al. also delivered the IL-2 immunoactivator together with DOX, but at a much lower dose. *N*,*N*,*N*-Trimethyl chitosan-based polymeric nanoparticles loaded with 2 mg/kg of DOX and 1.2 µg/animal of IL-2 were injected intravenously into SMMC 7721-bearing mice every two days. When further modified with 8.45% *w*/*w* folic acid, the polymeric co-delivery system resulted in a five-fold smaller tumor volume than those treated with DOX alone after 14 days [43].

Combination of IL-2 and PCT also has produced a synergistic effect in several reported studies. Wang et al. delivered IL-2 at 5 µg in combination with 10 µg of PCT per animal using P(MDS)-co-CES polymeric nanoparticles. Co-delivery of IL-2 and PCT resulted in a 2.5-fold lower tumor volume after 17 days of treatment than with individual drug loaded nanoparticles [102]. In another study, Zhao et al. delivered an IL-2 and PCT combination of 2.5 µg/kg and 10 mg/kg, respectively, in PLGA/Pluronic F127-based nanoparticles. The in vivo study using a murine melanoma B16-F10 cell line showed significant tumor growth and metastasis inhibition. Additionally, a prolonged overall survival rate was demonstrated in tumor-bearing mice treated by co-delivery of IL-2 and PCT [172].

The release profile of ILs is critical in designing co-delivery systems for IL and chemotherapeutic agents. Systemic administration of IL-2 may initiate an auto-immune response, resulting in side effects from the therapy. Previously, combination of local administration of IL-2 and systemic administration of PCT was used as a combination strategy [179]. IL-2 loaded in polymeric nanoparticles generated a controlled release profile in the systemic circulation. Co-encapsulation of IL-2 with chemotherapeutic agents not only induced a synergistic effect, but also minimized the auto-immune response. Although the co-delivery approach using a single delivery system offers many advantages, pharmacokinetics and cytotoxic side effect still need to be considered. The release profile of IL-2 is critical for the design of co-delivery systems for IL-2 and chemotherapeutic agents.

## 5. Conclusions and Perspectives

In this review, we have discussed the different types of block co-polymers that have been formulated into polymeric co-delivery systems for cancer treatment. We also focused on the different drug combinations used in co-delivery systems and the influence of the combinations on their therapeutic outcomes. As discussed earlier, targeting two multidrug resistance mechanisms simultaneously with a single delivery platform is a promising strategy that can provide synchronized pharmacokinetics and doses to the same cell population. Many co-delivery systems have been developed that exhibit promising anti-tumor efficacy, especially in multidrug resistant tumors. Nevertheless, it has been well-established that cancer cells have the inherent ability to avoid cell death by activation of various anti-apoptotic pathways. These pathways seem to be cell type specific and assault type specific. Therefore, it appears that the effectiveness of decreasing cell viability by double sensitization is cell-specific. That means a certain combination of drugs may work perfectly in one cell line, but may not work at all in another cell line without modification of the combination of drugs. A “one type fit all” formulation seems unlikely for eradication of all types of tumors clinically. Individualized therapy designed specifically for each type of cancer cell with different drug combination may be more likely to be necessary for an effective therapeutic outcome [180].

Polymeric nanoparticles are ideal platforms for co-delivery because of the multicompartments they contain. Amphiphilic co-polymers integrated with different properties have been synthesized to encapsulate hydrophilic agents as well as hydrophobic agents. Cationic moieties in co-polymers also provide the possibility for complexed nucleic acid molecules. Although there is much research focusing on combination delivery using polymeric nanoparticles, little emphasis has been put on the optimization of drug ratios to promote synergistic effects. The synergistic effect of polymeric co-delivery systems depends on multiple factors. First of all, it is important to ensure that the chemistries of delivery, carrier and therapeutic agents do not interact detrimentally with each other [86]. Another challenge is to ensure that the presence of one therapeutic agent does not interfere with the action of others. Additionally, the ratio between two therapeutic agents can directly determine the outcome of a co-delivery system. The release profile of small molecule drug and nucleic acid molecules is another major challenge that requires considerable research.

Overall, a deeper understanding of the optimal ratio between therapeutic agents and the natural heterogenicity of the tumor is necessary for development of polymeric co-delivery systems that maximize therapeutic effects. Additionally, multifunctional polymeric nanoparticles, especially stimuli-sensitive polymeric nanoparticles, allow high drug loading, optimized release profiles, and enhanced in vivo stability for the co-delivery of distinctly different classes of therapeutic molecules. There is a need to continue efforts at understanding the relationship between the mechanism of action of encapsulated therapeutic agents and their pharmacological activities. Resolving these challenges should result in multifunctional polymeric nanoparticles with significantly enhanced therapeutic efficacy.

## Figures and Tables

**Table 1:** Types of stimuli-sensitive polymers commonly used as co-delivery systems.

Types	Polymers	Dosage Form *	Drug 1	Drug 2	Cell Line	Ref.
pH-sensitive	PDEA-PDMA-PEG	M	siBcl-2	DOX	Hep G2	[41]
	PEG-PLL-PAsp	NP	siBcl-2	DOX	Hep G2	[42]
	Trimethyl Chitosan	NP	IL-2	DOX	SMMC7721	[43]
	PEI-PLA/PEG-PAsp	NP	siSurvivin	PCT	A549	[44]
	PEO-b-PCL	M	siMDR-1	DOX	MDA-MB-435	[45]
	PDP-PDHA	NP	shSurvivin	DOX	MCF7/ADR	[46]
Redox-sensitive	PEI-CD	NP	TRAIL pDNA	DOX	SKOV-3	[47]
	PEG2k-CLV-Dox/ miRNA-34a-S-S-PE	MM	miRNA-34a	DOX	MCF7, HT1080	[48]
	mPEG-PCL-SS-DOX/mPEG-PCL-SS-DTX	MM	DOX	DTX	MCF7	[49]
	Poly(acrylic acid)	H	DOX	CDDP	MCF7/ADR	[50]
	Gambogic acid-poly(amido amine)s	M	DTX	MMP9shRNA	MCF7	[51]
	PEG-PLG-PDMAPMA	NP	siMDR-1	DOX	MCF7	[52]
Thermo-sensitive	DH_700k_MF-13.5/MDocLF	HMM	DOX	DTX	CT-26	[53]
	DHmPEG-b-PELG	H	IL15	CDDP	B16F0-RFP	[54]
	PLGA–PEG–PLGA	H	DOX, MTX	CDDP	Saos-2, MG-63	[55]
	PLGA-DOX/PEO–PPO–PEO	NP	DOX	IFNγ	B16F10	[56]
	PECT	HM	DOX	131I	Hep G2	[57]
MMP-sensitive	PEG2k-CLV-Dox/ miRNA-34a-S-S-PE	MM	miRNA-34a	DOX	MCF7, HT1080	[48]
	PEG-pp-PEI-DOPE	M	siSurvivin	PCT	A549 T24	[58]
	PEG-PLA, G0–C14	NP	VEGF siRNA	PCT	HT-1080, A375, PC-3	[59]
Magnetic-responsive	PLGA/TPGS/OA	NP	TPGS	DOX	MCF7, MCF7/ADR	[60]
	ASA-MNPs-CDDP/mPEG-PLL-FA	NP	CDDP	TFPI2 DNA	HNE-1, NP69	[61]
	PCL/P(NIPAAm-co-HEMA-co-MAA-co-TMSPMA)	NP	DOX	MTX	MCF7	[62]

* MM—mixed micelles; M—micelles; NP—nanoparticles; H—hydrogels; HM—micelles entrapped in hydrogel, HMM—mixed micelles entrapped in hydrogel. PECT—poly(ε-caprolactone-co-1,4,8-trioxa[4.6]spiro-9-undecanone)-poly(ethyleneglycol)-poly(ε-caprolactone-co-1,4,8-trioxa[4.6]spiro-9-undecanone).

**Table 2:** Combinations of chemotherapeutics delivered in polymeric co-delivery systems.

Polymers	Drug 1	Concentration 1	Drug 2	Concentration 2	Cell line	Ref.
**In vitro**						
HA	CPT	0.05–5.0 μM	DOX	0.22–0.5 μM	BT-474,	[81]
HA	CPT	0.04–0.45 μM	DOX	0.02–0.4 μM	bEnd.3	[81]
PLL-PTX ^1^, HA30k-GEM ^2^	GEM	1 × 10^−4^–1.0 mM	PCT	1 × 10^−4^–1.0 mM	SCK HuCCT1	[121]
PEG-soyPC-PLGA)	DOX	1–50.0 ng/mL	triptolide (TPL)	0.05–1 folds over DOX	KB	[122]
PEG-PLGA	CUR	5–15 μM	Chrysin	15–45 μM	Caco-2	[123]
DOX-PEG-GEM	DOX	0.001–100 μM	GEM	0.001–100 μM	SKOV-3, MCF-7, MDA-MB-231	[124]
TPGS-PAE ^3^	DOX	0.031–1.0 μM	CUR	0.312–10 μM	SMMC7721	[115]
PEG-P(Glu)-P(Phe) ^4^	PCT	0.0041–3.0 µg/mL	CDDP	0.94–60 µg/mL	HeLa, A549	[125]
PSn(P2VP-b-(PAA-g-PNIPAM)) ^5^	PCT	1–15 µg/mL	camptothecin	1–15 µg/mL	A549	[126]
PLGA	PCT	10 μM	tetrandrine	10 μM	A2780	[127]
Polyphosphazene	DOX	12.5 μg/mL	CQ	1:1 and 2:1 over DOX	MCF7/ADR and HL60/ADR	[128]
mPEG-PLGA	DOX	Various ratios	PCT	Various ratios	A549, HepG2, B16	[79]
P(MeOx-b-BuOx-b-MeOx) ^6^	PCT	0–1 μg/mL	alkylated CDDP	0–2.5 μg/mL	LCC-6-MDR, A2780 A2780/CisR	[129]
PEG-P(Glu)-P(Phe)	PCT	CDDP/PCT = 10:1	CDDP	0–10 μg/ml	A2780	[130]
**In vivo**						
PHBV-PLGA	OXa	5 mg/kg	5-FU	25 mg/kg	CT26	[131]
HA	CPT	2 mg/kg	DOX	1.05 mg/kg	4T1	[81]
PLL-PTX, HA30k-GEM	GEM	108.8 ug/animal	PCT	54 ug/animal	HuCCT1	[121]
Pluronic F127-chitosan	DOX	25 mg/kg	PCT	20 mg/kg	Healthy rat	[118]
TPGS-PAE	DOX	1 mg/kg	CUR	10 mg/kg	SMMC 7721	[115]
PEG-P(Glu)-P(Phe)	PCT	3 mg/kg	CDDP	10 mg/kg	A549	[125]
Polyphosphazene	DOX	15 ng/animal	CQ	15 ng/animal	MCF7/ADR	[128]
PPBV ^7^	PCT	4 mg/kg	CUR	10 mg/kg	MCF7	[132]
P(MeOx-b-BuOx-b-MeOx)	PCT	20 mg/kg	alkylated CDDP	20 mg/kg	A2780/CisR	[129]
PEG-P(Glu)-P(Phe)	PCT	4 mg/kg	CDDP	4 mg/kg	A2780/Luc	[130]

^1^ Poly (l-lysine)–carboxylate paclitaxel. ^2^ Hyaluronic acid-gemcitabine. ^3^ d-α-tocopheryl poly(ethylene glycol) 1000-block-poly (β-amino ester). ^4^ Poly(ethylene glycol)-b-poly(l-glutamic acid)-b-poly(l-phenylalanine). ^5^ Star-graft quarterpolymers, composed of hydrophobic polystyrene and pH-sensitive poly(2-vinylpyridine)-b-poly(acrylic acid). ^6^ Poly(2-methyl-2-oxazoline-block-2-butyl-2-oxazoline-block-2-methyl-2-oxazoline). ^7^ Poly(ethylene glycol)-benzoic imine-poly(γ-benzyl-l-aspartate)-b-poly(1-vinylimidazole) block copolymer.

**Table 3:** Combinations of nucleic acids therapeutics and chemotherapeutics delivered in polymeric co-delivery systems.

Polymers	Drug 1	Concentration 1	Drug 2	Concentration 2	Cell Line	Ref.
**In vitro**						
P(MDS)-co-CES	TRAIL	10 nM	DOX	0.8 µM	SW480-TR	[157]
P(MDS)-co-CES	TRAIL	10 nM	PCT	1.67 µM	MCF7, T47D, MDA-MB-231	[158]
PEI-CD	TRAIL pDNA	2.5 µg/mL	DOX	0.5 µg/mL	SKOV-3	[47]
PLGA-PLA	p53 gene	2 µg/mL	DOX	0.9 µg/mL	Hep G2	[160]
PDMAEMA-PMPD	p53 gene	4 µg/mL	DOX	3 µg/mL	MCF7	[161]
PEI-PCL/FA-PEG-PGA	siBcl-2	20 nM	DOX	50 nM	Bel-7402	[96]
PEI-PCL/FA-PEG-PGA	siBcl-2	25 nM	DOX	0.5 µg/mL	C6	[162]
PDEA-PDMA-PEG	siBcl-2	20 nM	DOX	1.69 µM	Hep G2	[41]
PEG-PLL-PAsp	siBcl-2	100 nM	DOX	0.6 µg/mL	Hep G2	[42]
P(MDS)-co-CES	siBcl-2	20 nM	PCT	100/400 nM	MDA-MB-231	[102]
PDMAEMA-PCL-PDMAEMA	siBcl-2	188 nM	PCT	0.58 µM	PC3	[100]
PEI-CyD	shSurvivin	2 µg/mL	PCT	0.6 µg/mL	SKOV-3	[163]
PEG-pp-PEI-DOPE	siSurvivin	150 nM	PCT	12–24 nM	A549 T24	[58]
PEI-PLA/PEG-PAsp	siSurvivin	20 nM	PCT	0.096 µg/mL	A549	[44]
PEO-b-PCL	siMDR-1	100 nM	DOX	5 µg/mL	MDA-MB-435	[45]
PEI-DOPE	siMDR-1	100 nM	DOX	1 µg/mL	MCF7/ADR	[97]
PAMAM-PEG-DOPE	siMDR-1	125 nM	DOX	1.7 µg/mL	A2780/ADR	[91]
PAMAM-PEG-DOPE	siMDR-1	125 nM	DOX	0.43 µg/mL	MCF7/ADR	[91]
NSC-PLL-PA	siMDR-1	100 nM	DOX	5 µg/mL	Hep G2/ADM	[164]
PEG-PLG-PDMAPMA	siMDR-1	100 nM	DOX	3 µg/mL	MCF7	[52]
DMAB-PLGA	siMDR-1	100 nM	DOX	11.6 µg/mL	MCF7/ADR	[165]
HA/PEI-PLGA	miR-542-3p	100 nM	DOX	2 µg/mL	MDA-MB-231	[111]
PEG-PLGA-PLL	miR-7	100 nM	PCT	0.01 µg/mL	HO8910pm	[166]
PEG-PCC-GEM-DC-CAT	miR-205	100 nM	GEM	500 nM	MIA PaCa-2R, CAPAN-1R	[109]
PCL-PEG-PHIS	siVEGF	100 nM	PCT	2 µg/mL	MCF7	[167]
**In vivo**						
PEI-CD	TRAIL pDNA	30 µg/animal	DOX	6 µg/animal	SKOV-3	[47]
PAMAM-PEG-T7	pORF-hTRAIL	50 µg/animal	DOX	4 µg/animal	Bel-7402	[159]
PDMAEMA-PMPD	p53	1.5 mg/kg	DOX	2 mg/kg	MCF7	[161]
PEI-PCL/FA-PEG-PGA	siBcl-2	1.6 µg/kg	DOX	22.5 µg/kg	C6	[162]
PEG-PLL-PAsp	siBcl-2	200 µg/kg	DOX	1 mg/kg	Hep G2	[42]
mPEG-PCL-g-PEI	Msurvivin	5 mg/kg	DOX	4 mg/kg	B16-F10	[104]
PEI-CyD	shSurvivin	6 µg/animal	PCT	20 µg/animal	SKOV-3	[163]
P85-PEI/TGPS	shSurvivin	2 mg/kg	PCT	10 mg/kg	A549	[168]
PDP-PDHA	shSurvivin	2 mg/kg	DOX	6 mg/kg	MCF7/ADR	[169]
PEI-PLA/PEG-PAsp	siSurvivin	20 nM	PCT	0.096 µg/mL	A549	[44]
NSC-PLL-PA	siMDR-1	0.2 mg/kg	DOX	0.5 mg/kg	Hep G2/ADM	[164]
DOPE-PEI	siMDR-1	1.2 mg/kg	DOX	2 mg/kg	MCF7/ADR	[170]
POEG-st-Pmor	IL-36γ	50 µg/animal	DOX	5 mg/kg	4T1.2	[171]
Trimethyl Chitosan	IL-2	1.2 µg/animal	DOX	2 mg/kg	SMMC7721	[43]
P(MDS)-co-CES	IL-2	5 µg/animal	PCT	10 µg/animal	4T1	[102]
PLGA/Pluronic F127	IL-2	2.5 µg/kg	PCT	10 mg/kg	B16-F10	[172]
PEG-PLGA-PLL	miR-7	2 mg/kg	PCT	6 mg/kg	HO8910pm	[166]
PEG-PCC-GEM-DC-CAT	miR-205	1 mg/kg	GEM	40 mg/kg	MIA PaCa-2R	[109]
PCL-PEG-PHIS	siVEGF	5 mg/kg	PCT	1.2 mg/kg	MCF7	[167]
PEI-SA	siVEGF	0.3 mg/kg	DOX	0.45 mg/kg	Huh7	[98]
